# Calcitonin Gene-Related Peptide Monoclonal Antibodies Versus Botulinum Neurotoxin a in the Preventive Treatment of Chronic Migraine: An Adjusted Indirect Treatment Comparison Meta-Analysis

**DOI:** 10.3389/fphar.2021.671845

**Published:** 2021-05-19

**Authors:** Yao-Yao Chen, Xiao-Qian Ye, Tai-Chun Tang, Tian-Wei She, Min Chen, Hui Zheng

**Affiliations:** ^1^ The Third Hospital/Acupuncture and Tuina School, Chengdu University of Traditional Chinese Medicine, Chengdu, China; ^2^ The Rehabilitation College, Fujian University of Traditional Chinese Medicine, Fuzhou, China; ^3^ Chinese and Western Medicine Department of Clinical Medicine, North Sichuan Medical College, Nanchong, China; ^4^ Clinical Medicine School, Hospital of Chengdu University of Traditional Chinese Medicine, Chengdu, China

**Keywords:** CGRP monoclonal antibodies, botulinum neurotoxin A, indirect treatment comparison, chronic migraine, migraine prophylaxis

## Abstract

**Purpose:** Calcitonin gene-related peptide monoclonal antibodies (CGRPmAbs) are new agents approved by the US Food and Drug Administration for preventive treatment of chronic migraine. Comparison between CGRPmAbs and previously approved Botulinum neurotoxin A (BoNT-A) will inform optimal preventive treatment of chronic migraine, but head-to-head trials are lacking. We therefore aimed to perform adjusted indirect comparison between CGRPmAbs and BoNT-A through a meta-analysis.

**Methods:** OVID MEDLINE, EMBASE and the Cochrane central register of controlled trials, clinical registries, and government websites were searched from inception to September 2019. Randomized controlled trials comparing CGRPmAbs or BoNT-A with placebo in the preventive treatment of chronic migraine were included. The primary outcomes were headache days and migraine days measured at week 12. Data were synthesized by using a frequentist approach; and the treatments were ranked by P-score.

**Results:** We included 10 trials (*n* = 4,678) after screening 1049 candidates. Six trials were with low risk of bias. Fremanezumab had an effect similar to BoNT-A in the reduction of headache days at week 12 (standard mean difference [SMD] 0.08, 95%CI -0.55 to -0.7). Galcanezumab reduced more migraine days than BoNT-A at week 12 (SMD, -0.94, 95%CI −1.24 to −0.63); fremanezumab showed similar findings (SMD, −0.55, 95%CI −0.85 to −0.24). Galcanezumab and fremanezumab had better effect in mitigating headache impact at week 12. CGRPmAbs and BoNT-A had similar adverse event rate.

**Conclusion:** CGRPmAbs and BoNT-A had similar effect in the preventive treatment of chronic migraine. BoNT-A might be preferentially selected owing to its cost-effectiveness profiles. Further studies with direct comparison of the two treatments are warranted.

## Clinical Implications

• Direct comparison of calcitonin gene-related peptide monoclonal antibodies (CGRPmAbs) vs. botulinum neurotoxin A (BoNT-A) was lacking.
• CGRPmAbs and BoNT-A had similar effect in the preventive treatment of chronic migraine.
• CGRPmAbs and BoNT-A had similar adverse event rate.
• BoNT-A might be preferentially selected owing to its cost-effectiveness profiles.

## Introduction

Patients with chronic migraine have monthly headaches ≥15 days and monthly migraine attacks ≥8 days for at least 3 months (Headache Classification Committee of the International Headache Society (IHS), 2013). Chronic migraine affects about 2% of the general population and about 8% of patients with migraine. Compared with episodic migraine, chronic migraine has larger impact on socioeconomic aspect and quality of life (Buse et al., 2012). The annual cost for the management of chronic migraine is estimated to be fourfold higher than the cost for episodic migraine (Munakata et al., 2009); and chronic migraine is usually correlated to medication overuse headaches (Antonaci et al., 2016), which makes its management more complicated.

The treatment of chronic migraine includes two steps. The first is to stop or reduce the intake of acute analgesics to prevent medication overuse, and the second step is to use preventive treatment. Several pharmacological and non-pharmacological treatments are suggested for the preventive treatment of chronic migraine, but very few of them are evidence based. In pharmacological treatments, topiramate is the only orally administered drug with high-quality evidence to support its efficacy and safety in treating chronic migraine, specifically. However, the high rate of adverse events and the potential risk of causing depression restrict its use for chronic migraine. There is a lack of high-quality evidence for non-pharmacological treatments.

Botulinum neurotoxin A (BoNT-A) is the first treatment that is specifically approved for chronic migraine by the US Food and Drug Administration (FDA), and its efficacy was confirmed in two large-scale trials (Aurora et al., 2010; Diener et al., 2010) and their subsequent secondary analyses (Aurora et al., 2011; Lipton et al., 2011; Aurora et al., 2014; Diener et al., 2014). Its safety was recently examined in a study with real-life and longer-term design^11^. BoNT-A is therefore the main preventive treatment for chronic migraine. In recent years, many studies showed the promising effect of calcitonin gene-related peptide monoclonal antibodies (CGRPmAbs) in the treatment of migraine; and several large-scale studies showed the efficacy and safety of CGRPmAbs (Silberstein et al., 2017; Detke et al., 2018; Dodick et al., 2019). On the basis of these studies, FDA approved erenumab, fremanezumab, and galcanezumab in preventive treatment of migraine; and UK National Health Service recommended the use of fremanezumab in the preventive treatment of chronic migraine.

Which is the optimal selection for the preventive treatment of chronic migraine? The clinical uncertainty could be better resolved by providing evidence of the comparative effectiveness between CGRPmAbs and routinely practiced BoNT-A. However, no head-to-head trial with randomized design exists. Under the condition, indirect treatment comparison was proposed, which was assumed to provide effect estimates of comparison between two interventions that share one or more common comparators (Sutton et al., 2008).

The objective of this study was to compare CGRPmAbs with BoNT-A in the preventive treatment of chronic migraine through an adjusted indirect comparison meta-analysis.

## Methods

### Protocol and Registration

The protocol of this study was registered and published on PROSPERO (CRD42018089201) (She et al., 2020). The design and conduction of the study were in accordance with the Preferred reporting items for systematic reviews and meta-analyses (PRISMA) statement (Moher et al., 2009) and its extension for network meta-analysis (Hutton et al., 2015).

### Eligibility Criteria

We included randomized controlled trials (RCTs) comparing CGRPmAbs or BoNT-A with placebo in the treatment of chronic migraine. RCTs with N-of-one design or cross-over design were excluded, since the interventions may have long-term effects and the duration of the persistent effect was unclarified (Aurora et al., 2010; Giamberardino et al., 2016; Silberstein et al., 2017). We included RCTs that recruited chronic migraine participants according to the criteria developed by the International Headache Society. RCTs that recruited participants with both chronic migraine and episodic migraine were included only if they reported them separately. RCTs of erenumab for migraine prophylaxis were excluded because of its anti-receptor action. RCTs were included when at least one of the following outcomes was reported: mean monthly migraine days or migraine frequency; the monthly head-hours; the intensity of headache attack (using visual analog scale or other pain intensity rating scales); monthly frequency or amount of acute medication intake; specific assessment scales for migraine (the Six-item Headache Impact Test [HIT-6](Yang et al., 2011) and the Migraine Disability Assessment [MIDAS](Stewart et al., 2001)); adverse event rate or tolerability (defined as the number of dropouts owing to adverse effect).

### Study Source

We searched OVID Medline, embase, Cochrane register of clinical trials (CENTRAL) from inception to September 2019 without any language restriction, using search strategies developed in advance. Before developing search strategy, we performed a pilot search using keywords—botulinum toxin, CGRP, and trial. Three experienced reviewers (Y-YC, MC, and HZ) developed the search strategies, the rationale and the specific details of the stratgies were showed in Supplementary Appendix S1. Registries of clinical trials like clinicaltrials.gov were also searched for unpublished trials, and we tried to contact the authors of these unpublished trials for efficacy data. The website of Food and Drug Administration was searched, and the reference of the systematic reviews published in recent 5 years was read, to find out any missed trials. The records of potentially eligible studies were imported into Zotero (version 5.0), and two reviewers (Y-YC and T-WS) independently screened the titles and abstracts of the records, and disagreements on the eligibility of the trials were solved by group discussion. Full-text copies of potentially eligible RCTs were acquired for further evaluation, and then we obtained necessary information from the eligible RCTs.

### Data Extraction and Risk of Bias Assessment

Standardized data extraction form was designed by using Epi Info (version 7.2.2.6). The following information were extracted: trial characteristics (first author, year of publication, study sites, total sample size, diagnostic criteria, treatments and comparisons, primary analysis dataset, and main conclusion); participant’s characteristics (proportion of female, mean age, headache frequency at baseline, and disease duration); treatment details (dose, frequency, and duration of treatment); outcomes (definition of outcomes, assessment timepoints, and results of the outcomes). Two reviewers (X-QY and T-CT) independently extracted data from the included RCTs; the reviewers compared each data item they extracted, found out the difference between the two datasets, corrected extraction errors, and tidied into one dataset for analysis.

Risk of bias of the included trials was assessed by using the Cochrane risk of bias tool (Higgins and Green, 2008). RCTs were classified as having low risk of bias if none of the six domains (Random Sequence Generation, Allocation Concealment, Blinding of Participants and Researchers, Incomplete Outcome Data, Selective Reporting, Other Bias) was rated as high risk of bias and three or fewer were rated as unclear risk; moderate if one was rated as high risk of bias, or none was rated as high risk of bias but four or more were rated as unclear risk; and all other cases were assumed to have high risk of bias (Furukawa et al., 2019).

### Outcome Assessments

The primary outcomes were the mean change in monthly headache days and the mean change in monthly migraine days. The number of days with headache or migraine is recommended to be adopted as one of the primary outcomes for trials assessing the effect of an intervention for migraine prophylaxis (Tassorelli et al., 2018), and the effect of an intervention were commonly assessed every month; we therefore assessed these two outcomes as primary outcomes.

Secondary outcomes included total monthly headache hours, >50% reduction in headache frequency, HIT-6, MIDAS, and treatment-related adverse event rate. We used the total headache hours to assess the cumulative duration of headache attack in a month, since one headache day could be counted when a headache duration lasted longer than 4 h. Responder is normally defined as a >50% reduction in headache frequency in migraine prophylaxis trials, and we aimed to assess the responder rate in this review. HIT-6 and MIDAS are scales for measuring the health impact of migraine; higher scores indicate lower quality of life. Tolerability was measured by calculating the rate of dropouts owing to adverse effect.

### Data Synthesis

An adjusted indirect treatment comparison was performed on the basis of Bucher’s method (Bucher et al., 1997), which calculated the indirect comparison estimates of treatment A vs. treatment B (effectAB) by the difference between A and B in their relative effect to a common comparator C (effectAB = effectAC - effectBC). The variance of the effectAB was the sum of the variances of effectAC and effectBC according to Bucher’s method, we further adjusted the effectAB variance according to Rücker’s method since several trials were multi-arm studies; the Rücker’s method was a statistical model built on the basis of electrical network and graphical theory (Rücker, 2012). The advantage of this model lies in a combination of the Bucher’s method and the adjustment for multi-arm studies. We performed the analysis by using R (version 3.6.0, netmeta package) (Rücker, 2012).

We analyzed the effect size of CGRPmAbs (treatment A) or BoNT-A (treatment B) by comparing with placebo control (treatment C). Changes in monthly headache days, migraine days, total headache hours, HIT-6, and MIDAS were calculated as standardized mean differences (SMDs) and their relative 95%confidence intervals (95%CIs). >50% reduction in headache frequency, adverse event rate, and tolerability were calculated as relative ratios (RRs) and related 95%CIs. We applied a continuity correction for RCTs with a 0 cell count by adding 0.5 to all cell frequencies (Sankey et al., 1996).

We assessed between-study heterogeneity by Cochran’s Q test and further assessed the consistency of the analysis by using a design-by-treatment decomposition approach (Higgins et al., 2012). Global I^2^ statistics was also used to assess the extent of heterogeneity for each outcome measurement, which is roughly classified according to the Cochane handbook (version 5.1): 0–40%, might not be important; 30–60%, may represent moderate heterogeneity; 50–90%, may represent substantial heterogeneity; 75–100%: considerable heterogeneity. Analysis of heterogeneity was also performed by using netmeta packages in R 3.6.0.

We assessed the P-score of CGRPmAbs or BoNT-A, which measures the extent of certainty that a treatment is better than another (Rücker and Schwarzer, 2015) and is commonly used to rank treatments in a network meta-analysis. The significance level of the comparison between CGRPmAbs and BoNT-A was unavailable in this indirect treatment comparison, and a significant difference between them was defined as a 95%CI of RR or SMD excluding null value.

Considering the potential heterogeneity in the administration ways and timepoints of CGRPmAbs, we performed a subgroup analysis by including the treatment arms of CGRPmAbs that adopted commercially available dosage and recommended treatment intervals (fremenezumab was administered quarterly at a dose of 675 mg, galcanezumab was administered 120 mg monthly, and eptinezumab was administered 100 mg quarterly). The primary outcomes and responde rate were preferentially analyzed for efficacy outcomes, since these outcomes were the reason for designing CGRPmAbs.Considering the equivalence of baseline characteristics, we performed a sensitivity analysis by including two BoNT-A trials and three CGRPmAbs trials that had similar baseline characteritics and re-analyzing the primary outcomes.

## Results

### Trial Characteristics

We included 10 RCTs (Ondo et al., 2004; Freitag et al., 2008; Aurora et al., 2010; Diener et al., 2010; Sandrini et al., 2011; Hollanda et al., 2014; Silberstein et al., 2017; Detke et al., 2018; Dodick et al., 2019; Pijpers et al., 2019) (*n* = 4,678) after screening 1049 articles (Supplementary Appendix S2). Trials characteristics were shown in Table 1. Six RCTs were multi-center design, and 3 of them were multi-national design. The multi-center RCTs were conducted in United States and Italy, respectively. Study duration ranged from 12 to 68 weeks. One RCT (Hollanda et al., 2014) recruited chronic migraine patients with cephalic cutaneous allodynia; two (Sandrini et al., 2011; Pijpers et al., 2019) recruited patients with medication overuse headache; two (Freitag et al., 2008; Hollanda et al., 2014) recruited patients without medication overuse headache, and six (Ondo et al., 2004; Aurora et al., 2010; Diener et al., 2010; Silberstein et al., 2017; Detke et al., 2018; Dodick et al., 2019) recruited both types of patients. Four RCTs (Aurora et al., 2010; Diener et al., 2010; Silberstein et al., 2017; Detke et al., 2018) recruited patients with or without aura; one RCT (Sandrini et al., 2011) recruited patients with chronic migraine without aura. Two BoNT-A trials adopted ICHD-I or revised ICHD criteria (Ondo et al., 2004; Freitag et al., 2008), four adopted ICHD-II(Aurora et al., 2010; Diener et al., 2010; Sandrini et al., 2011; Hollanda et al., 2014), and one adopted ICHD-3 beta criteria (Pijpers et al., 2019); the three CGRPmAbs trials adopted ICHD-3 beta criteria (Silberstein et al., 2017; Detke et al., 2018; Dodick et al., 2019). The mean baseline headache days were 19.8–25.3 days per month in the BoNT-A trials and were 20.4–21.4 days per month in the CGRPmAbs trials. All except two trials (Freitag et al., 2008; Hollanda et al., 2014) recruited patients with medication overuse.

**TABLE 1:** Characteristics of the included trials.

Study ID	Sample size(n)	Country (study sites)	Diagnostic criteria	Age (yr)	Female (%)	Migraine history (yr)	Baseline headache days (sd/range)	Medication overuse (%)	Study duration (wk)	Treatment	Treatment details	Primary outcome	Adverse events
Freitag (2008)	41	United States (1)	ICHD-I	42.3	73.17	NA	23 (16–28)	No (0%)	16	BoNT-A (*n* = 20) vs. placebo (*n* = 21)	BoNT-A administrated 100U/session for one session over 4 months	Migraine episodes	Stiff neck, sinus infection, hair loss, amenorrhea
Aurora et al. (2010)	679	United States (56)	ICHD-II	41.7	87.50	20.45	19.8 (3.6)	Yes (68%)	56	BoNT-A (*n* = 341) vs. placebo (*n* = 338)	BoNT-A or placebo administrated 155–195U/session for one sessions over 3 months	Headache days	NA
Diener et al. (2010)	705	Germany (56)	ICHD-II	41	85.40	18.05	19.9 (3.7)	Yes (63%)	56	BoNT-A (*n* = 347) vs. placebo (*n* = 358)	BoNT-A/placebo administrated 155–195U/session for one session over 16 weeks	Headache days	NA
Sandrini et al. (2011)	56	Italy (3)	ICHD-II	48.75	80.36	20	24.8 (5)	Yes (100%)	24	BoNT-A (*n* = 27) vs. placebo (*n* = 29)	BoNT-A/placebo administrated 100U/session for one session over 3 months	Headache days	Neck pain, pain at the site of injection, Muscular weakness
Silberstein et al. (2017)	1130	United States (132)	ICHD-3 beta	41.3	88.00	19.8	20.4 (4.1)	Probabaly yes (95%)	12	Fremanezumab 675 mg (*n* = 376) Fremanezumab 675/225/225 mg (n = 379) vs. placebo (n = 375)	Fremanezumab/placebo administered for a total of 3 sessions over 3 months	Headache days	Muscular weakness, neck pain, neck rigidity, injection-site pain, hypertonia, headache, shoulder/arm pain, and hypesthesia
Detke et al. (2018)	1085	United Kingdom (116)	ICHD-3 beta	41	85.00	21.1	21.4 (4.1)	Yes (64%)	68	Galcanezumab 120 mg (*n* = 273) Galcanezumab 240 mg (*n* = 274) vs. placebo (*n* = 538)	Galcanezumab/placebo for a total of 3 sessions over 3 months	Migraine days	Injection-site pain, nasopharyngitis, upper respiratory tract infection, injection-site erythema, fatigue, back pain, urinary tract infection, abdominal pain, neck pain
Dodick et al. (2019)	616	United States (92)	ICHD-3 beta	37	87.00	NA	21.2 (3.9)	Yes (51.9%)	53	Eptinezumab 300 mg (*n* = 121) Eptinezumab 100 mg (*n* = 122) Eptinezumab 30 mg (*n* = 122) Eptinezumab 10 mg (*n* = 130) vs. placebo (*n* = 121)	Eptinezumab/placebo administrated for one session over 3 months	≥75% decrease in monthly migraine days	Upper respiratory tract infection, dizziness, nausea, nasopharyngitis, sinusitis, bronchitis, migraine
Hollanda et al. (2014)	38	Brazil (1)	ICHD-II	45.3	76.30	NA	NA	No (0%)	38	BoNT-A (*n* = 20) vs. placebo (*n* = 18)	BoNT-A/placebo administrated 24U/session for one session over 12 weeks	Frequency of headache episodes with allodynia	Pain in injected points, burning sensation in injected points, headache after injection
Ondo et al. (2004)	58	United States (1)	Revised HIS criteria	47	81.70	NA	25.3(NA)	Yes (53.3%)	29	BoNT-A (*n* = 29) vs. placebo (*n* = 29)	BoNT-A/placebo administrated 200U/session for one session over 12 weeks	Headache-free days	NA
Pijpers et al. (2019)	179	Netherland (1)	ICHD-3 beta	45.2	76.00	27.6	21.4 (4.8)	Yes (100%)	16	BoNT-A (*n* = 90) vs. placebo (*n* = 89)	BoNT-A/placebo administrated 155U/session for three sessions over 12 weeks, placebo administrated (17.5 units BTA + saline)/session for a total of 3 sessions over 12 weeks	Headache days	Pain, small hematoma at injection sites

Abbreviations: ICHD, International Classification of Headache Disorders. BoNT-A, botulinum neurotoxin A.

Annotations: ICHD-I.

Seven RCTs had a low risk of bias in randomization sequence generation; eight RCTs had a low risk of bias in allocation concealment; nine RCTs had a low risk of bias in blinding; six RCTs had a low risk of bias in selective reporting; and all the RCTs were at a low risk of presenting incomplete outcome data. Six RCTs had low risk of bias and four had moderate risk in the overall assessment of risk of bias (Supplementary Appendix S3).

### Headache Days

**FIGURE 1:**
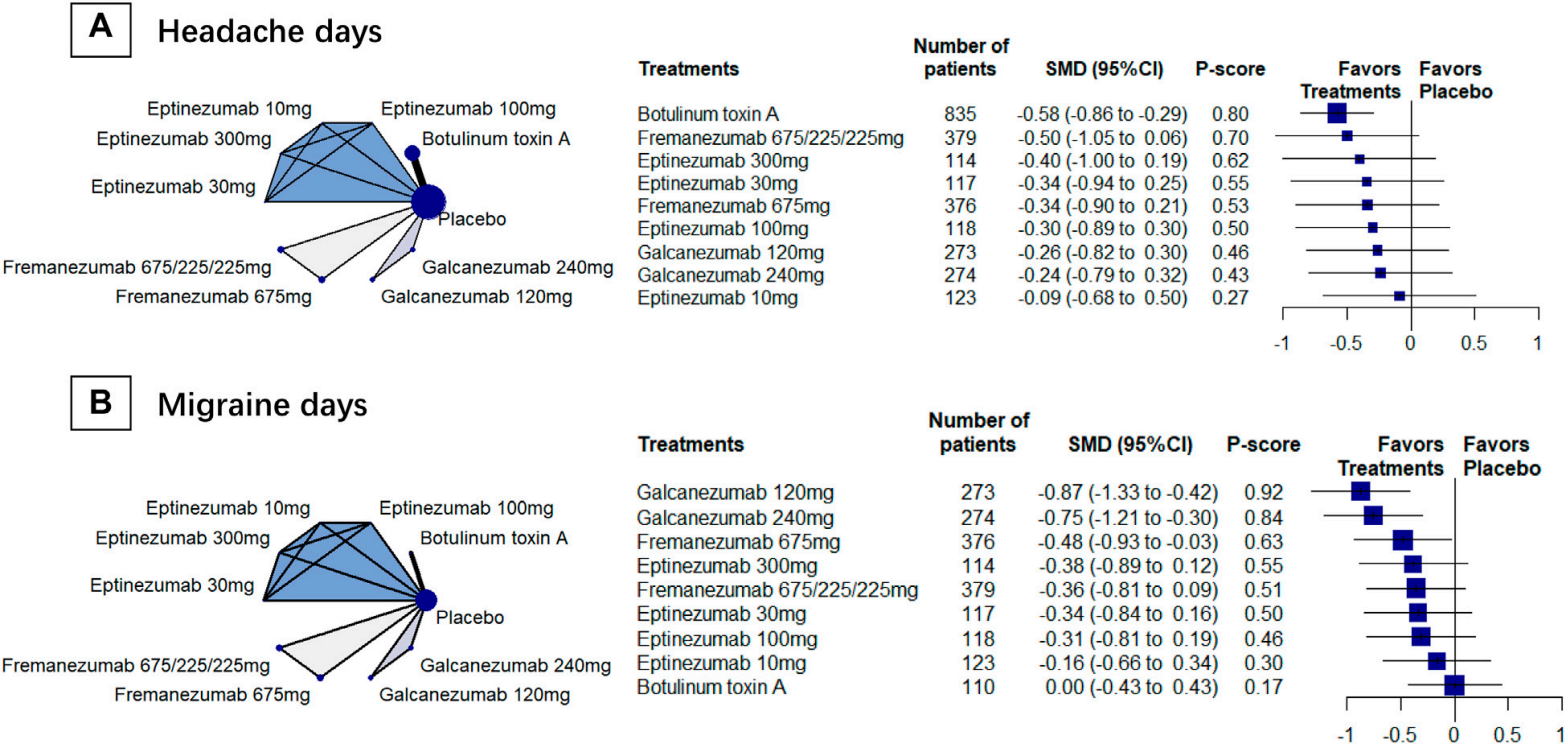
The primary outcomes; *Abbreviation*: 95% CI, 95% confidence interval. Fremanezumab 675/225/225 mg, fremanezumab was injected 675 mg at baseline, 225 mg at week 4, and another 225 mg at week 8. SMD, standardized mean difference. *Footnotes*: The figure shows the results of the primary outcomes: **(A)** monthly headache days and **(B)** monthly migraine days. The left of both **(A)** and **(B)** shows the geometry of the networks, and the right shows the forest plots using placebo as a reference comparator. The size of the blue nodes corresponds to the number of participants allocated to treatments. Direct comparison was linked by a line between two treatments; the thickness of the lines corresponds to the number of trials that studied the treatment. The blue or gray triangle among treatments indicates a three-arm design of an RCT. The treatments were ranked by P-scores. A P-score is an estimation of the mean probability of a treatment to be the best treatment. A treatment with the highest P-score ranked the most effective. A SMD>0 indicates superiority of a treatment over placebo.

We included 6 RCTs (*n* = 2809) in week-12 assessment, and the results showed that BoNT-A was the most effective (SMD, −0.58 [95%CI, −0.86 to −0.29]; P-score = 0.79, Figure 1A). Fremanezumab had similar effect to BoNT-A (Table 2). We found considerable heterogeneity in the analysis (global I^2^ = 83%), and we found that the heterogeneity originated from the design of BoNT-A vs. placebo (Cochran’s Q = 23.54, *p* < 0.001). Similar results were found in the week-8 assessment (Supplementary Appendix S4).

**TABLE 2:** Compared with BoNT-A in outcomes.

	Fremanezumab	Galcanezumab	Eptinezumab
Headache days			
12w	675/225/225 mg; SMD, 0.08 [95%CI, -0.55 to 0.70]	120 mg; SMD, 0.31 [95%CI, -0.31 to 0.94]	100 mg; SMD, 0.28 [95%CI, -0.38 to 0.94]
12w	675 mg; SMD; 0.23 [95%CI, -0.39 to 0.86]	240 mg; SMD, 0.34 [95%CI, -0.29 to 0.96]	300 mg; SMD, 0.17 [95%CI, -0.49 to 0.83]
Migraine days			
12w	675/225/225 mg; SMD, -0.36 [95%CI, -0.99 to 0.27]	120 mg; SMD, -0.87 [95%CI, -1.50 to -0.24]	100 mg; SMD, -0.31 [95%CI, -0.97 to 0.35]
12w	675 mg; SMD, -0.48 [95%CI, -1.10 to 0.15]	240 mg; SMD -0.76 [95%CI, -1.38 to -0.13]	300 mg; SMD, -0.38 [95%CI, -1.05 to 0.28]
Headache hours			
24w	NA	120 mg; SMD, -4.48 [95%CI, -4.80 to -4.17]	NA
24w	NA	240 mg; SMD -3.51 [95%CI, -3.79 to -3.23]	NA
>50% reduction in headache frequency			
12w	NA	120 mg; RR, 1.25 [95%CI, 0.27 to 5.79]	10 mg; RR, 0.70 [95%CI, 0.15 to 3.21]
12w	NA	240 mg;RR, 1.25 [95%CI, 0.27 to 5.77]	30 mg,RR, 0.84 [95%CI, 0.18 to 3.88]
12w	NA	NA	100 mg;RR, 0.81 [95%CI, 0.18 to 3.72]
12w	NA	NA	300 mg; RR, 0.93 [95%CI, 0.20 to 4.26]
HIT-6			
12w	675/225/225 mg; SMD -4.46 [95%CI, -6.02 to -2.91]	NA	10 mg; RR, 0.25 [95%CI, -1.30 to 1.80]
12w	675 mg; SMD, -3.82 [95%CI, -5.37 to -2.28]	NA	30 mg; RR, 0.25 [95%CI, -1.30 to 1.80]
12w	NA	NA	100 mg; RR, 0.14 [95%CI, -1.41 to 1.69]
12w	NA	NA	300 mg; RR, -0.70 [95%CI, -2.25 to 0.85]
MIDAS			
12w	NA	120 mg; SMD, -1.88 [95%CI, -2.20 to -1.56]	NA
12w	NA	240 mg; SMD, -1.00 [95%CI, -1.31 to -0.70]	NA
Treatment-related adverse event			
12w	675/225/225 mg; RR, 1.17 [95%CI, 0.79 to 1.74]	120 mg; RR, 1.73 [95%CI, 0.60 to 5.05]	NA
12w	675 mg; RR, 1.13 [95%CI, 0.76 to 1.68]	240 mg; RR, 1.68 [95%CI, 0.58 to 4.89]	NA

Abbreviations: 95% CI, 95% confidence interval. Fremanezumab 675/225/225mg, fremanezumab was injected 675 mg at baseline, 225 mg at week 4, and another 225 mg at week 8. HIT-6, Headache Impact Test. MIDAS, Migraine Disability Scale. RR, relative ratio. SMD, standardized mean difference.

### Migraine Days

We included 4 RCTs (*n* = 2452) in week-12 assessment, and the results showed that galcanezumab had the largest reduction in migraine days (SMD, −0.87 [95%CI, −1.33 to −0.42]; P-score = 0.90; Figure 1B). Compared with BoNT-A, galcanezumab 240 mg (SMD, −0.76 [95%CI, −1.38 to −0.13]) and galcanezumab 120 mg (SMD, −0.87 [95%CI, −1.50 to −0.24]) showed significantly more reduction in migraine days (Table 2). Fremanezumab 675/225/225 mg (SMD, −0.36, [95%CI, −0.99 to 0.27]) and fremanezumab 675 mg (SMD, −0.48 [95%CI, −1.10 to 0.15]) showed similar results (Table 2). Moderate heterogeneity was found in the analysis (global I^2^ = 42.6%; Cochran’s Q = 1.74, *p* = 0.187). The assessments at week 4 and week 8 presented similar results (Supplementary Appendix S4).

### Headache Hours

**FIGURE 2:**
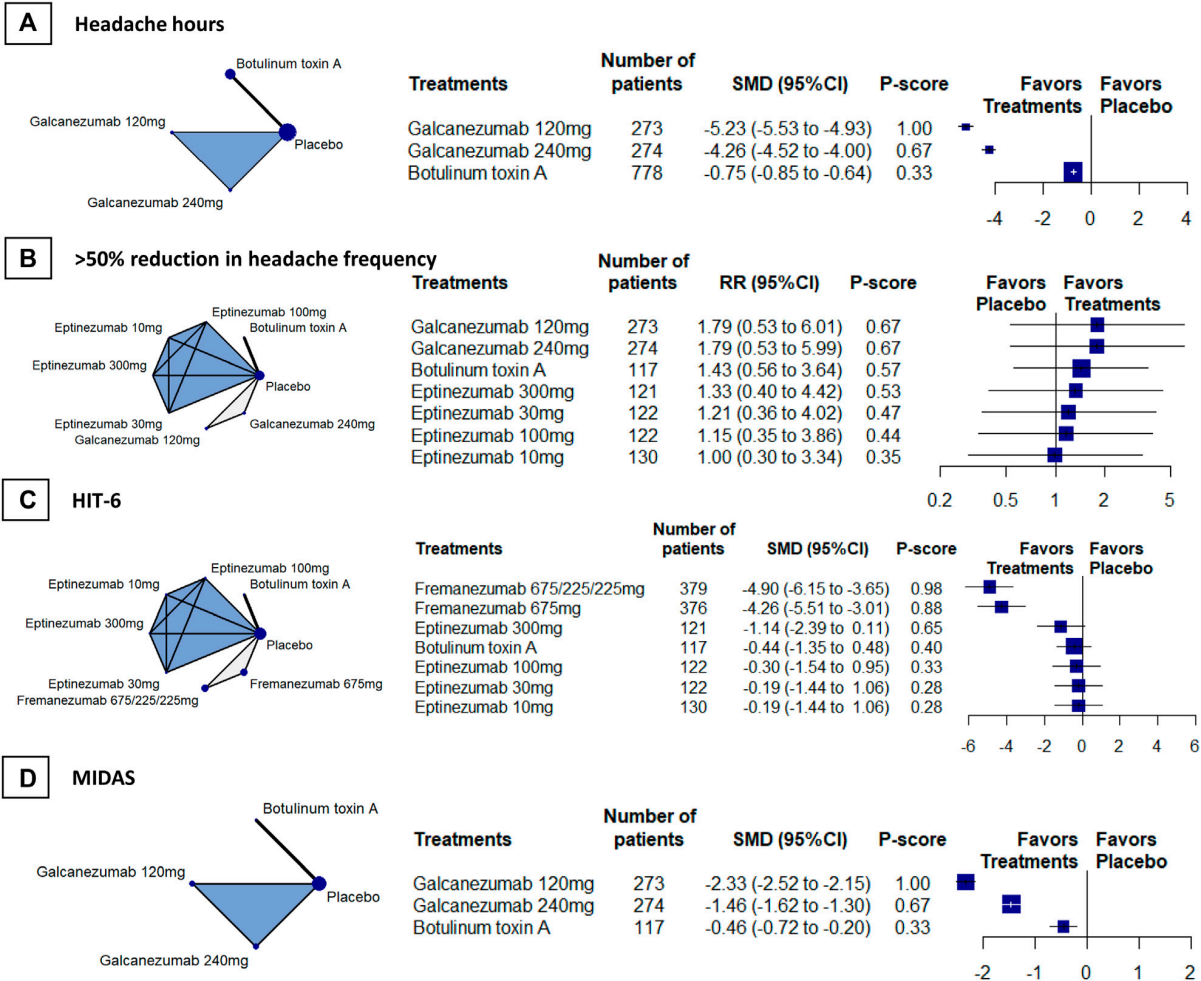
Secondary efficacy outcomes. *Abbreviations*: 95% CI, 95% confidence interval. Fremanezumab 675/225/225 mg, fremanezumab was injected 675 mg at baseline, 225 mg at week 4, and another 225 mg at week 8. HIT-6, Headache Impact Test. MIDAS, Migraine Disability Scale. RR, relative ratio. SMD, standardized mean difference. *Footnotes*: The figure shows the results of the secondary efficacy outcomes: **(A)** headache hours **(B)** > 50% reduction in headache frequency, **(C)** HIT-6, and **(D)** MIDAS. The left of **(A)**, **(B) (C)**, and **(D)** shows the geometry of the networks, and the right shows the forest plots using placebo as a reference comparator. The size of the blue nodes corresponds to the number of participants allocated to treatments. Direct comparison was linked by a line between two treatments; the thickness of the lines corresponds to the number of trials that studied the treatment. The blue or gray triangle among treatments indicates a three-arm design of an RCT. The treatments were ranked by P-scores. A P-score is an estimation of the mean probability of a treatment to be the best treatment. A treatment with the highest P-score ranked the most effective. An RR > 1 indicates superiority of a treatment over placebo.

We included 4 RCTs (*n* = 2742) in week-24 assessment. The meta-analysis showed that galcanezumab 120 mg group ranked the most effective (SMD, −5.23, 95%CI [–5.53 to−4.93; P-score = 1.00; Figure 2A). Galcanezumab 120 mg (SMD, −4.48 [95%CI, −4.80 to −4.17]) and galcanezumab 240 mg (SMD, −4.26 [95%CI, −4.52 to −4.00]) had significantly larger reduction in headache hours that BoNT-A (Table 2). No important heterogeneity was found in the analysis (global I^2^ = 4.1%; Cochran’s Q = 2.09, *p* = 0.352).

### >50% Reduction in Headache Frequency

We included 4 RCTs (*n* = 1826) in week-12 assessment, the result showed that galcanezumab 120 mg (RR, 1.79, 95%CI [0.53 to 6.01]; P-score = 0.67; Figure 2B) ranked the most effective, but no difference was found between galcanezumab and BoNT-A (Table 2). Considerable heterogeneity was found in the analysis (global I^2^ = 79.3%), and the heterogeneity originated from the design of BoNT-A vs. placebo (Cochran’s Q = 4.83, *p* = 0.028). The responder rates in the placebo arms were similar in BoNT-A (29%, 95%CI 21–39%) and CGRPmAbs (28%, 95%CI 12–52%).

### HIT-6

We included 4 RCTs (*n* = 1981) in week-12 assessment, and the results showed that fremanezumab 675/225/225 mg ranked the most effective (SMD, –4.90 [95% CI, −6.15 to −3.65]; P-score = 0.98; Figure 2C). Fremanezumab 675/225/225 mg (SMD, −4.46 [95% CI, −6.02 to−2.91]) and fremanezumab 675 mg (SMD, −3.82 [95%CI, −5.37 to −2.28]) had significantly better effect than BoNT-A in reducing HIT-6 score (Table 2). Considerable heterogeneity was found in the analysis (global I^2^ = 88.4%), and the heterogeneity originated from the design of BoNT-A vs. placebo (Cochran’s Q = 8.59, *p* = 0.003).

### MIDAS

We included 3 RCTs (*n* = 1320) in week-12 assessment, the results showed that galcanezumab 120 mg ranked the most effective (SMD, −2.33 [95% CI, −2.52 to −2.15]; P-score = 1.00; Figure 2D). Galcanezumab 120 mg (SMD, −1.88 [95%CI, −2.20 to −1.56]) and galcanezumab 240 mg (SMD, −1.00 [95% CI, −1.31 to −0.70]) were superior over BoNT-A in reducing MIDAS score (Table 2). No important heterogeneity was found in the analysis (global I^2^ = 0%; Cochran’s Q = 0.91, *p* = 0.339) and insignificant heterogeneity was found.

### Treatment-Related Adverse Events

**FIGURE 3:**
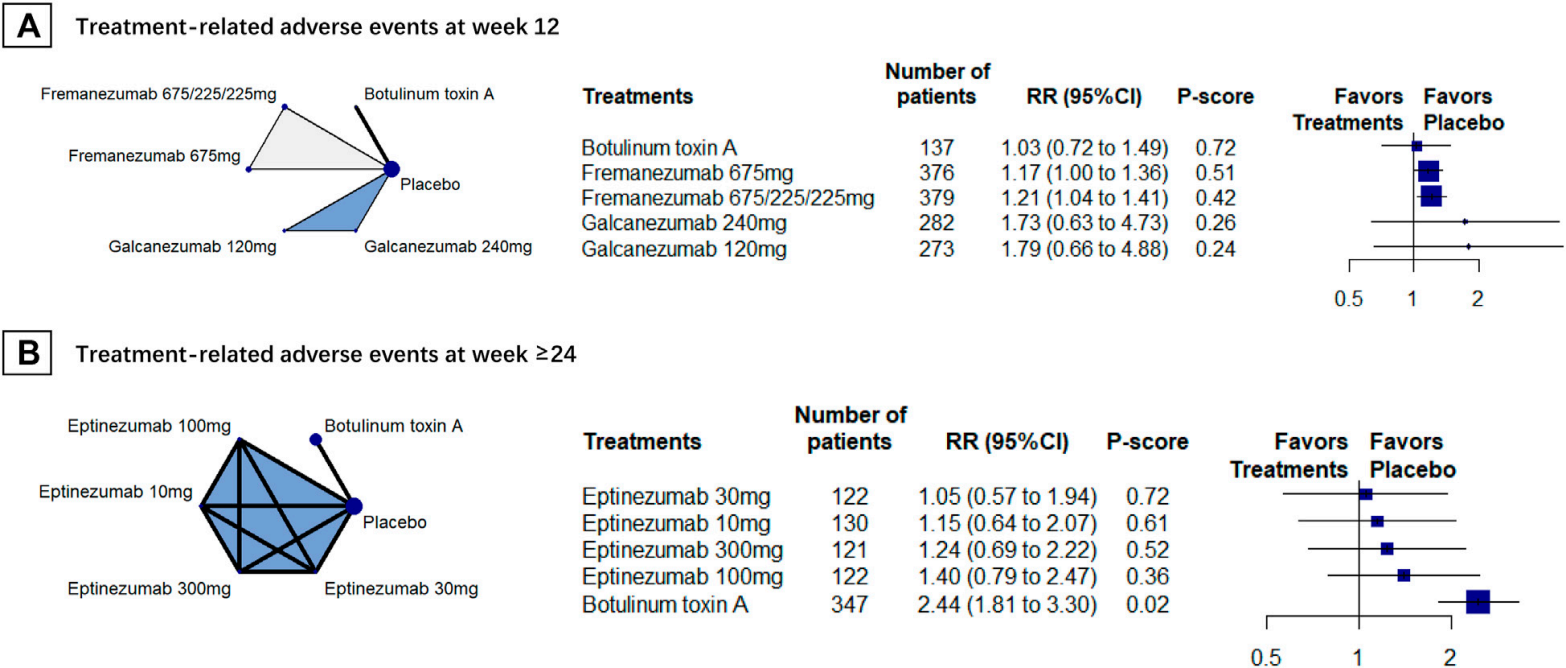
Treatment-related adverse events; *Abbreviations*: 95% CI, 95% confidence interval. Fremanezumab 675/225/225 mg, fremanezumab was injected 675 mg at baseline, 225 mg at week 4, and another 225 mg at week 8. RR, relative ratio. *Footnotes*: The figure shows the results of treatment-related adverse events at **(A)** week 12 and **(B)** week 24. The left of **(A)** and **(B)** shows the geometry of the networks, and the right shows the forest plots using placebo as a reference comparator. The size of the blue nodes corresponds to the number of participants allocated to treatments. Direct comparison was linked by a line between two treatments; the thickness of the lines corresponds to the number of trials that studied the treatment. The blue or gray triangle among treatments indicates a three-arm design of an RCT. The treatments were ranked by P-scores. A P-score is an estimation of the mean probability of a treatment to be the best treatment. A treatment with the least adverse events had the highest P-score. An RR > 1 indicates higher adverse event rate of a treatment over placebo.

We included 5 RCTs (*n* = 2516) in week-12 assessment. The results showed that BoNT-A caused the least treatment-related adverse events (RR, 1.03, 95% CI 0.72 to 1.49, P-score = 0.72; Figure 3A). However, no difference found between BoNT-A and CGRPmAbs (Table 2). No heterogeneity was found in the analysis (global I^2^ = 0%; Cochran’s Q = 0.87, *p* = 0.647).

Two RCTs (*n* = 1321) were included for the week-24 assessment, and the results showed that eptinezumab 30 mg had the least treatment-related adverse events (RR, 1.05 [95%CI, 0.57 to 1.94]; P-score = 0.72; Figure 3B). Eptinezumab 10 mg (RR, 0.47 [95%CI, 0.24–0.91]) and eptinezumab 30 mg (RR, 0.43 [95%CI, 0.22–0.85]) had significantly fewer treatment-related adverse events than BoNT-A. Heterogeneity results in this analysis was unavailable because of few studies were included.

### Subgroup Analysis

The subgroup analysis in headache days included 4 RCTs (*n* = 2314); the results showed that BoNT-A was the most effective but it was no significantly superior over fremanezumab 675 mg quartely (BoNT-A vs. fremanezumab, SMD −0.03 [95%CI, −0.27 to 0.22]). The subgroup analysis in migraine days included 5 RCTs (*n* = 3145); the results showed that galcanezumab 120 mg monthly was the most effective but it was not significantly superior over fremenezumab 675 mg quartely and BoNT-A (Supplementary Appendix S5). The subgroup analysis in responder rate included 4 RCTs (*n* = 1289); the results showed that galcanezumab 120 mg monthly was the most effective, but it was not significantly superior over eptinezumab 100 mg quartely and BoNT-A. The subgroup analyses showed results consistent with the main analysis (Supplementary Appendix S5).

### Sensitivity Analysis

The sensitivity analysis (Supplementary Appendix S6) showed that fremenezuma 675/225/225 mg (SMD, −0.5 [95%CI, −0.71 to −0.29]; P-score = 0.88) and BoNT-A (SMD, −0.4 [95%CI, −0.56 to −0.25]; P-score = 0.72) were the most effective treatments in reducing monthly headache days; and galcanezumab 120 mg (SMD, −0.87 [95%CI, −1.14 to −0.6]; P-score = 0.97) was the most effective treatment in reducing monthly migraine days. The results were consistent with the main analysis.

## Discussion

### Summary of Findings

We performed an adjusted indirect treatment comparison meta-analysis aiming to compare CGRPmAbs with BoNT-A in the preventive treatment of chronic migraine. We found that: 1) CGRPmAbs and BoNT-A were both effective in reducing headache days. Galcanezumab and fremanezumab were superior over BoNT-A in reducing migraine days at week 12, and galcanezumab was superior over BoNT-A in reducing headache hours at week 24, which indicates a short-term superiority of CGRPmAbs over BoNT-A. However, a contradictory finding in >50% reduction in headache frequency showed that there was no difference between galcanezumab and BoNT-A. 2) CGRPmAbs and BoNT-A were both effective in reducing HIT-6 and MIDAS. Fremanezumab was superior over BoNT-A in reducing HIT-6 scores at week 12, galcanezumab had better effect than BoNT-A in reducing MIDAS at week 12, which also indicates a short-term advantage of CGRPmAbs. 3) Both CGRPmAbs and BoNT-A caused similar adverse event rate, and the tolerability rate between them was also similar. There was little difference between them at week 12 but CGRPmAbs (eptinezumab) had lower adverse event rate at week 24.

### Comparison of CGRPmAbs and BoNT-A

Equivalence in baseline characteristics is the basis of the comparison between CGRPmAbs and BoNT-A. The diagnostic criteria of chronic migraine are mainly ICHD-II in BoNT-A trials—especially the two trials (Aurora et al., 2010; Diener et al., 2010) with the largest sample size and the largest weight in the meta-analysis, and the CGRPmAbs trials adopted ICHD-3 beta criteria. The difference between ICHD-II and the ICHD-3 beta lies in that ICHD-3 beta requires additionally having monthly migraine days for at least 8 days. The two largest BoNT-A trials both reported their mean baseline migraine days—19 days per month, so we assummed that the monthly headache days and monthly migraine days were comparable between BoNT-A and CGRPmAbs trials. In addition, the two BoNT-A trials had similar proportion of participants with medication overuse as the three CGRPmAbs trials. Our sensitivity analyses including the five trials (Aurora et al., 2010; Diener et al., 2010; Silberstein et al., 2017; Detke et al., 2018; Dodick et al., 2019) showed consistent results with the main analysis, which confirmed our findings.

One consideration in the comparatability of CGRPmAbs vs. BoNT-A was the response rate of placebo. Owing to the difference in ways of administration, placebo response might vary between BoNT-A and CGRPmAbs. A study recently reported that the placebo BoNT-A had a higher responder rate than the placebo CGRPmAbs (Kokoti et al., 2020), however, our study found them similar. We found that it might be the consequence of including BoNT-A trials (Sandrini et al., 2011; Pijpers et al., 2019) with lower placebo response rate than the two large-scale BoNT-A trials (Aurora et al., 2010; Diener et al., 2010) that showed a placebo response of 35%, which also indicated that the response rate of placebo would change across different populations and study settings.

Another consideration in the comparatability was that, unlike BoNT-A that had a definite and univocal injection paradigm, CGRPmAbs presented with different ways of administration (eg, monthly or quartely administration); and CGRPmAbs were tested in dosages that were not used in practice. We therefore performed a subgroup analysis to include CGRPmAbs with commercially available or recommended dosage, and similar results were found with the main analysis, which might indicate that commercially available CGRPmAbs and BoNT-A had similar effects in reducing headache days. The subgroup analysis might also indicate that difference in administration ways was not the main source of heterogeneity of the meta-analysis.

We had an interesting finding in the study—although CGRPmAbs and BoNT-A had similar effects on the reduction of headache days, CGRPmAbs caused more reduction in migraine days than BoNT-A. Inconsistent findings between headache days and migraine days were found; the difference in effect may be caused by their difference in biological mechanism. The anti-migraine effect of BoNT-A is associated with relaxation of pathological muscle tension, anti-inflammatory effect, and affecting central afferent transport—includes inhibiting the release of substance P and CGRP (Ramachandran and Yaksh, 2014). The release of CGRP and the location of its receptor are closely related to trigeminovascular system (Edvinsson et al., 2018). Peripheral actions in migraine are associated with trigeminal CGRP and its roles in vasodilation, neurogenic inflammation, and peripheral sensitization (Russo, 2015); CGRPmAbs theoretically might have stronger effect in migraine headaches than BoNT-A. Their biological mechanisms partly explain the difference in reducing migraine days in our finding—indicates that CGRPmAbs might be preferable for patients with higher frequency of migraine attacks. Another explanation for this finding might be the difference in baseline migraine days—we calculated the change-from-baseline values for migraine days, and the trial with a higher baseline value is more likely to have larger changes in migraine days. However, we found that the migraine days were 16–19 days in three large-scale CGRP trials (Silberstein et al., 2017; Detke et al., 2018; Dodick et al., 2019) and 19 days in two large-scale BoNT-A trials (Aurora et al., 2010; Diener et al., 2010)—indicates that the baseline value might be an unimportant factor in the difference of changes in migraine days.

Although CGRPmAbs showed some short-term benefits in reducing migraine days and headache hours, there are still concerns about its advantages in the preventive treatment of chronic migraine. First, some headache experts would quibble about differentiating between headache days and migraine days. The headaches are milder and resemble tension-type headaches, and they might be actually mild migraines. Making this distinction is probably not useful. Second, Contradicting findings were found across outcomes—CGRPmAbs showed advantages in migraine days and headache hours but no advantages in the >50% reduction in headache frequency. These contradictory findings might indicate heterogeneous definition in the outcomes of headache frequency—headache days, migraine days, migraine frequency, and migraine episodes. Third, CGRPmAbs take action immediately after several days of administration, while the action of BoNTA is often delayed, so CGRPmAbs might not had advantages in the long-term when compared with BoNT-A. Based on these grounds, we concluded that CGRPmAbs and BoNT-A had similar effect in the preventive treatment of chronic migraine. The advantages of CGRPmAbs against BoNT-A should be further examined in head-to-head comparison trials.

### Clinical Relevance

With the advantage in reducing migraine days, CGRPmAbs were also superior over BoNT-A in reducing headache hours and headache impact (HIT-6 and MIDAS). The superiority of CGRPmAbs over BoNT-A was found based on the results of indirect comparisons. Whether to apply the evidence to clinical practice should be considered in several aspects. First, the confidence of the indirect comparison was a major concern. Although we found moderate heterogeneity in the analysis of migraine days (one of the primary outcomes) and no important heterogeneity in headache hours, and MIDAS, we found considerable heterogeneity in headache days, >50% reduction in headache frequency, and HIT-6. The design-by-treatment analysis by decomposing of Cochran’s Q found that the heterogeneity was from the design of BoNT-A vs. placebo. This finding was consistent with the results of a recently published systematic review (Shen and Wang, 2020). Significant heterogeneity in the design of BoNT-A vs. placebo might be the consequence of variations in the injection protocol of BoNT-A, although this hypothesis could not be confirmed in previous studies. Second, we performed a traditional contrast-based meta-analysis, in which the effect size of an intervention may vary as the effect size of its control changes. Linde’s study showed the placebo effect of different treatments varied significantly (Meissner et al., 2013), and a recent systematic review showed that the response rate to CGRPmAbs placebo was 23.6 vs. 36.4% in BoNT-A placebo—showing a difference as large as 13% (Kokoti et al., 2020). These findings indicated that a head-to-head comparison between CGRPmAbs and BoNT-A may still be warranted. Third, two large PREEMPT trials estimated that, to avoid one day with headache attack, the cost of BoNT-A was GBP 18 (Herd et al., 2018), while the cost of CGRPmAbs is higher (Kendall and Enright, 2012). It will place a greater financial burden on migraine patients. Most of the outcomes were assessed at week 12—the long-term effectiveness of CGRPmAbs was still under investigation.

Both CGRPmAbs and BoNT-A are with mild adverse effect that were transient and no additional medical care was needed. Their adverse events were similar—most of them were neck pain and injection-site pain; these adverse effects are closely related to the administration instead of the actual effect of drug. Other adverse effects might be the consequence of the actual effect of the drugs; eg, some patients have a feeling of muscle weakness after BoNT-A injection, and some reported hypertonia or infections after CGRPmAbs injection. Although eptinezumab appeared to cause significantly less adverse events than BoNT-A at week 24, BoNT-A was still preferable since most of the adverse events were mild and tolerable.

### Limitations

Our study has several limitations. First, we compared CGRPmAbs and BoNT-A indirectly by using placebo as a common comparator; the variations in the placebo effect sizes and heterogeneity between BoNT-A trials might influence our results. Such as the doses of BoNT-A varied from 20 to 200U, and the injection sites varied between studies—although many of the trials followed the injection protocol that were used in two large scale trials (Aurora et al., 2010; Diener et al., 2010), which suggests that the difference between CGRPmAbs and BoNT-A might be overestimated. In addition, the diversity in study population may also contribute to the heterogeneity of the study, and the ethnicity might play an important role. However, most of the included studies reported no information on ethnicity, which makes this assumption unverified. Second, the number of trials included was insufficient, which has impact on the accuracy of the effect estimates and P-score calculation.

## Conclusion

In summary, although CGRPmAbs showed some advantages in reducing migraine days and a possibly small advantage in causing less adverse events, BoNT-A might be preferentially selected owing to its cost-effectiveness profiles. Our study results also indicated that Head-to-head comparison trials with long-term assessments are warranted to verify the study findings.

## Data Availability

The raw data supporting the conclusions of this article will be made available by the authors, without undue reservation.
