# Supplementary material for: Calcitonin Gene-Related Peptide Monoclonal Antibodies Versus Botulinum Neurotoxin a in the Preventive Treatment of Chronic Migraine: An Adjusted Indirect Treatment Comparison Meta-Analysis
Source: Front Pharmacol. 2021 May 19;12:671845. doi: 10.3389/fphar.2021.671845 (PMC8170150; doi:10.3389/fphar.2021.671845)
Supplement: Supplementary file 1 [file DataSheet1.docx]

**Appendix**

[Appendix A. Search strategy 2](#_Toc70081281)

[Appendix B. Study flowchart 4](#_Toc70081282)

[Appendix C. Risk-of-bias assessment 5](#_Toc70081283)

[Appendix D. Outcomes assessed at other timepoints 6](#_Toc70081284)

[Appendix E. Subgroup analysis 12](#_Toc70081285)

[Appendix F. Sensitivity analysis 15](#_Toc70081286)

# Appendix A. Search strategy

**OVID MEDLINE**

1. randomised controlled trial.pt.
2. randomized controlled trial.pt.
3. controlled clinical trial.pt.
4. randomized.ab.
5. randomised.ab.
6. randomly.ab.
7. or/1-6
8. limit 7 to humans
9. exp Migraine Disorders/
10. exp Migraine without Aura/
11. exp Migraine with Aura/
12. chronic migraine.tw.
13. chronic migraine.ab.
14. chronic migrain$.ab.
15. or/9-14
16. exp Botulinum Toxins, Type A/
17. Botulinum toxin$.ab.
18. BOTOX.ab.
19. Onabotulinumtoxin.ab.
20. exp Receptors, Calcitonin Gene-Related Peptide/
21. Calcitonin Gene-Related Peptide.ab.
22. CGRP.ab.
23. Or/16-22
24. 7 AND 15 AND 23

**EMBASE**

1. ‘Randomized Controlled Trial’/exp
2. ‘Randomized Controlled Trials as Topic’/exp
3. ‘randomized controlled trial’:ab,ti
4. ‘controlled clinical trial’/exp
5. ‘controlled clinical trial’:ab,ti
6. (#1 OR #2 OR #3 OR #4 OR #5) AND [humans]/lim
7. ‘Migraine Disorders’/exp
8. ‘Migraine without Aura’/exp
9. ‘Migraine with Aura’/exp
10. ‘chronic migraine’:ab,ti
11. ‘chronic migraine’:ab,ti
12. ‘chronic migrain$’:ab,ti
13. #7 OR #8 OR #9 OR #10 OR #11 OR #12
14. ‘Botulinum Toxins, Type A’/exp
15. ‘Botulinum toxin$’:ab,ti
16. ‘BOTOX’:ab,ti
17. ‘Onabotulinumtoxin’:ab,ti
18. ‘Receptors, Calcitonin Gene-Related Peptide’/exp
19. ‘Calcitonin Gene-Related Peptide’:ab,ti
20. ‘CGRP’:ab,ti
21. #14 OR #15 OR #16 OR #17 OR #18 OR #19 OR #20
22. 6 AND 13 AND 21

**CENTRAL (Cochrane Central Register of Controlled Trials)**

#1 "randomised controlled trial"

#2 "randomized controlled trial"

#3 "controlled clinical trial"

#4 #1 or #2 or #3

#5 "chronic migraine"

#6 "Botulinum Toxin"

#7 "Botulinum Toxins"

#8 "BOTOX"

#9 "Onabotulinumtoxin"

#10 "Calcitonin Gene-Related Peptide"

#11 "CGRP"

#12 #6 or #7 or #8 or #9 or #10 or #11

#13 #4 and #5 and #12

# Appendix B. Study flowchart

**Figure B1. Study flowchart**

# Appendix C. Risk-of-bias assessment

| Study ID | Sequence  generation | Allocation  concealment | Blinding  participant | Blinding_  therapist | Blinding_  assessor | Unpublished data | Selective  report | Attrition  bias |
| --- | --- | --- | --- | --- | --- | --- | --- | --- |
| Freitag, 2007 | Unclear risk of bias | Unclear risk of bias | Low risk of bias | Low risk of bias | Unclear risk of bias | Low risk of bias | Low risk of bias | High risk of bias |
| Aurora, 2010 | Low risk of bias | Low risk of bias | Low risk of bias | Low risk of bias | Low risk of bias | Low risk of bias | Low risk of bias | Low risk of bias |
| Diener, 2010 | Low risk of bias | Low risk of bias | Low risk of bias | Low risk of bias | Low risk of bias | Low risk of bias | Low risk of bias | Low risk of bias |
| Sandrini, 2011 | Low risk of bias | Low risk of bias | Low risk of bias | Low risk of bias | Low risk of bias | Low risk of bias | Low risk of bias | High risk of bias |
| Silberstein, 2017 | Low risk of bias | Low risk of bias | Low risk of bias | Low risk of bias | Low risk of bias | Low risk of bias | Low risk of bias | Low risk of bias |
| Detke, 2018 | Low risk of bias | Unclear risk of bias | Low risk of bias | Low risk of bias | Low risk of bias | Low risk of bias | Low risk of bias | Low risk of bias |
| Dodick, 2019 | Low risk of bias | Unclear risk of bias | Low risk of bias | Low risk of bias | Unclear risk of bias | High risk of bias | High risk of bias | Low risk of bias |
| Hollanda, 2014 | Low risk of bias | Low risk of bias | Low risk of bias | Low risk of bias | Low risk of bias | Low risk of bias | Low risk of bias | Low risk of bias |
| Ondo, 2004 | Unclear risk of bias | Unclear risk of bias | Low risk of bias | Low risk of bias | Unclear risk of bias | Low risk of bias | Low risk of bias | Low risk of bias |
| Pijpers, 2019 | Low risk of bias | Low risk of bias | Low risk of bias | Low risk of bias | Unclear risk of bias | Low risk of bias | Low risk of bias | Low risk of bias |

# Appendix D. Outcomes assessed at other timepoints

**D1. Headache days assessed at week 8**


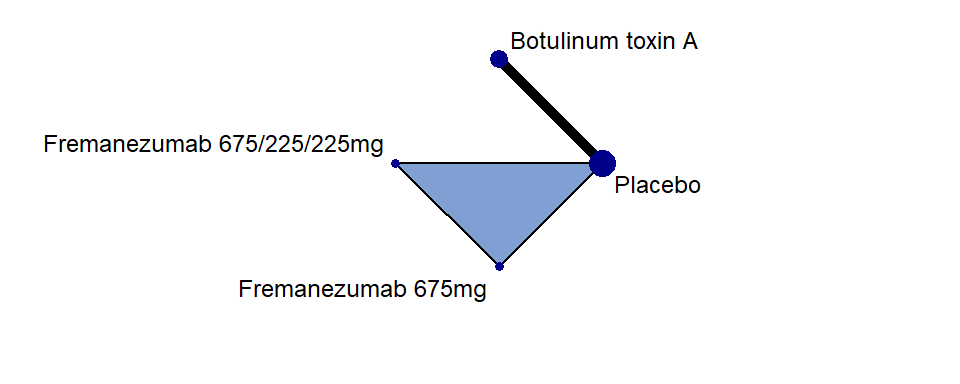


**Figure D1. Network geometry of** **headache days assessed at week 8**

The size of the blue nodes corresponds to the number of participants allocated to treatments. Direct comparison was linked by a line between two treatments; the thickness of the lines corresponds to the number of trials that studied the treatment. The blue or grey triangle among treatments indicates a three-arm design of an RCT.


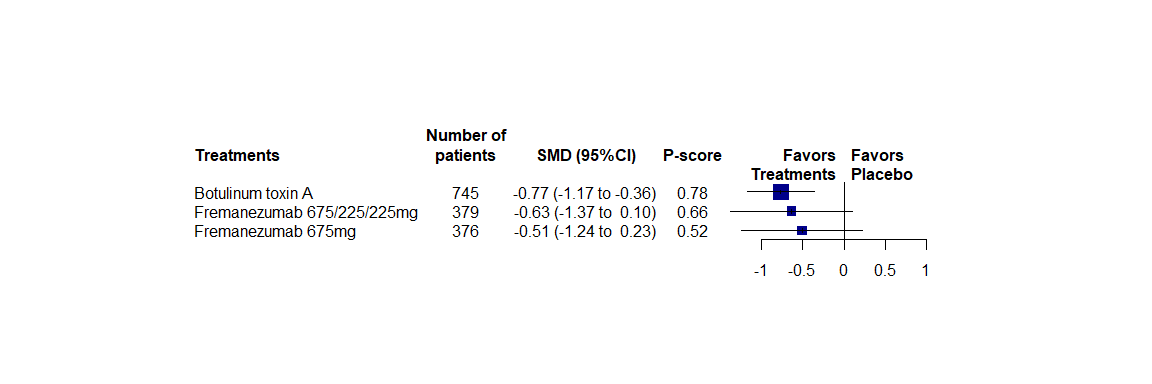


**Figure D2.** **Forest plot of headache days assessed at week 8**

95%CI, 95% confidence interval. SMD, standardized mean difference.

The forest plot showed the effect sizes of the treatments using placebo as a reference comparator. The treatments were ranked by P-scores. A P-score is an estimation of the mean probability of a treatment to be the best treatment. A treatment with the highest P-score ranked the most effective. A SMD>0 indicates superiority of a treatment over placebo.

**D2. Migraine days assessed at week 4**


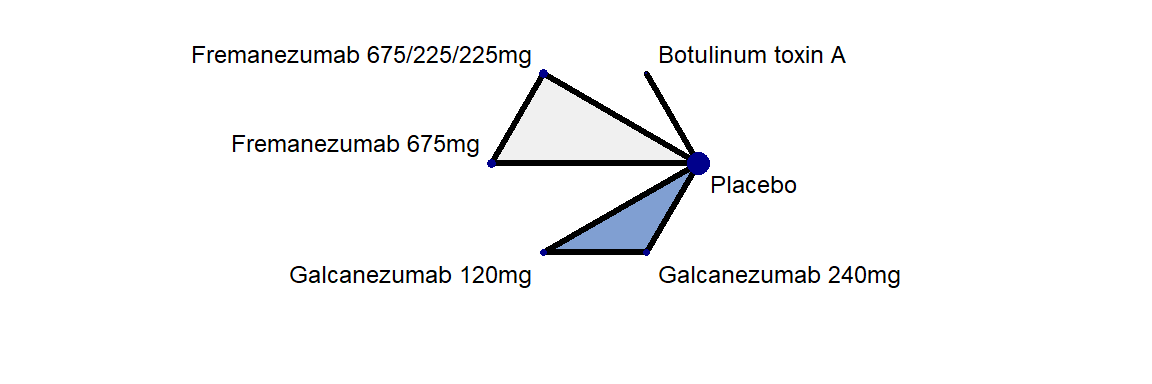


**Figure D3. Network geometry of migraine days assessed at week 4**

The size of the blue nodes corresponds to the number of participants allocated to treatments. Direct comparison was linked by a line between two treatments; the thickness of the lines corresponds to the number of trials that studied the treatment. The blue or grey triangle among treatments indicates a three-arm design of an RCT.


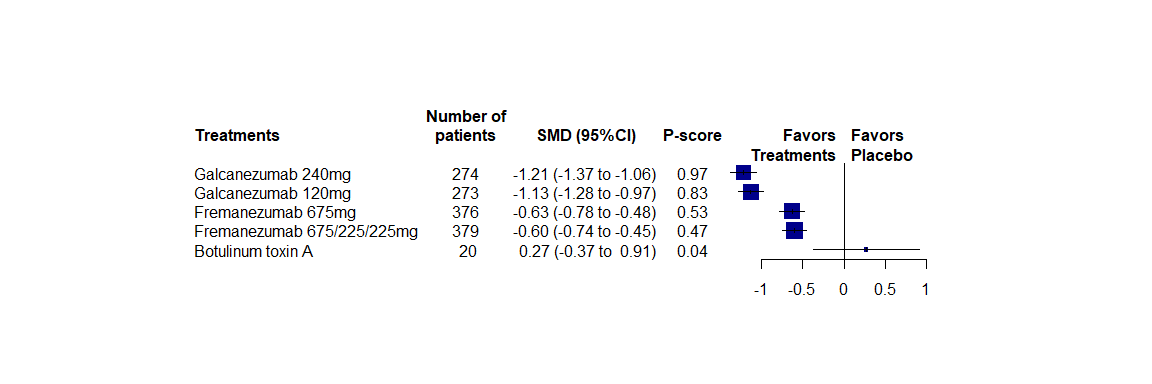


**Figure D4. Forest plot of migraine days assessed at week 4**

95%CI, 95% confidence interval. SMD, standardized mean difference.

The forest plot showed the effect sizes of the treatments using placebo as a reference comparator. The treatments were ranked by P-scores. A P-score is an estimation of the mean probability of a treatment to be the best treatment. A treatment with the highest P-score ranked the most effective. A SMD>0 indicates superiority of a treatment over placebo.

**D3. Migraine days assessed at week 8**


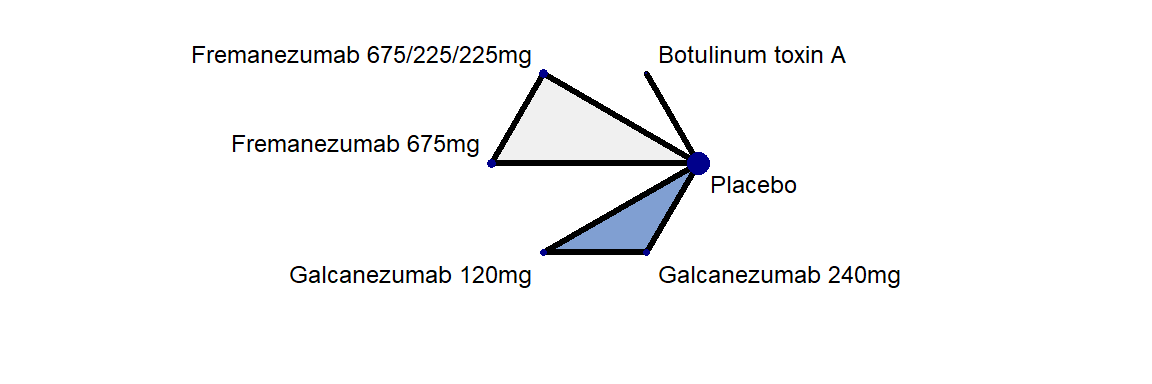


**Figure D5. Network geometry of migraine days assessed at week 8**

The size of the blue nodes corresponds to the number of participants allocated to treatments. Direct comparison was linked by a line between two treatments; the thickness of the lines corresponds to the number of trials that studied the treatment. The blue or grey triangle among treatments indicates a three-arm design of an RCT.


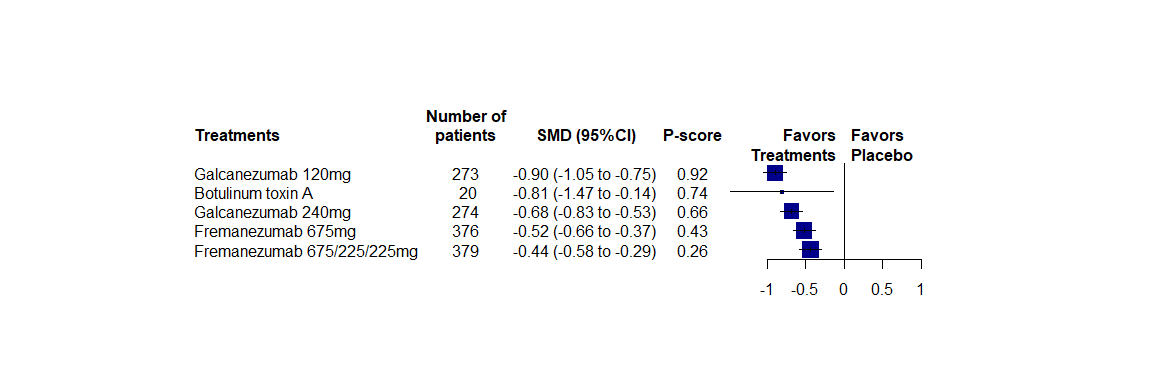


**Figure D6. Forrest plot of migraine days assessed at week 8**

95%CI, 95% confidence interval. SMD, standardized mean difference.

The forest plot showed the effect sizes of the treatments using placebo as a reference comparator. The treatments were ranked by P-scores. A P-score is an estimation of the mean probability of a treatment to be the best treatment. A treatment with the highest P-score ranked the most effective. A SMD>0 indicates superiority of a treatment over placebo.

# Appendix E. Subgroup analysis

**E1. Headache days**

**
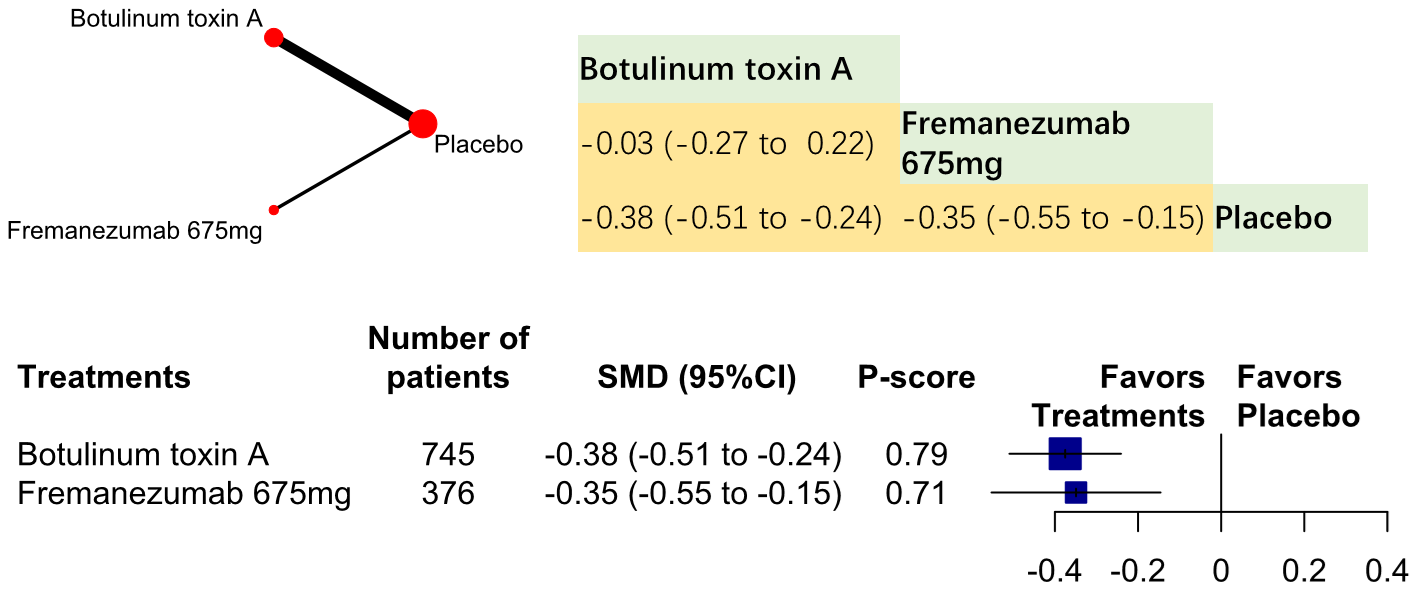
**

**Figure E1. Subgroup analysis in headache days**

The subgroup analysis included the treatment arms of CGRPmAbs that adopted commercially available dosage and recommended treatment intervals (fremenezumab was administered quarterly at a dose of 675mg, galcanezumab was administered 120mg monthly, and eptinezumab was administered 100mg quarterly). The results showed that BoNT-A and fremanezumab 675mg quartely had similar effect in reducing headache days (BoNT-A versus fremanezumab, SMD -0.03[95%CI, -0.27 to 0.22]).

**E2. Migraine days**

**
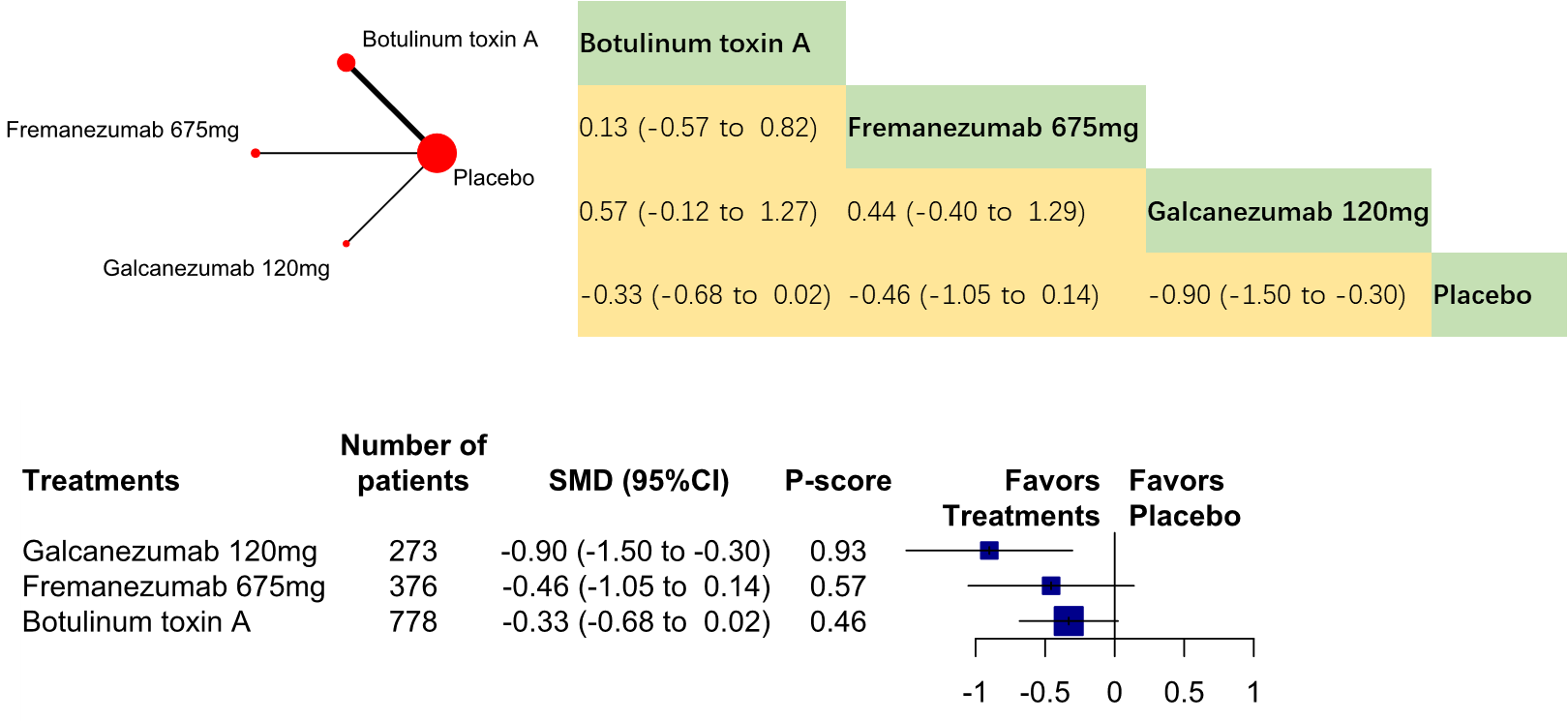
**

**Figure E2. Subgroup analysis in migraine days**

The subgroup analysis included the treatment arms of CGRPmAbs that adopted commercially available dosage and recommended treatment intervals (fremenezumab was administered quarterly at a dose of 675mg, galcanezumab was administered 120mg monthly, and eptinezumab was administered 100mg quarterly). The results showed that galcanezumab 120mg monthly was the most effective but it was not significantly superior over fremenezumab and BoNT-A.

**E3. Responder rate**


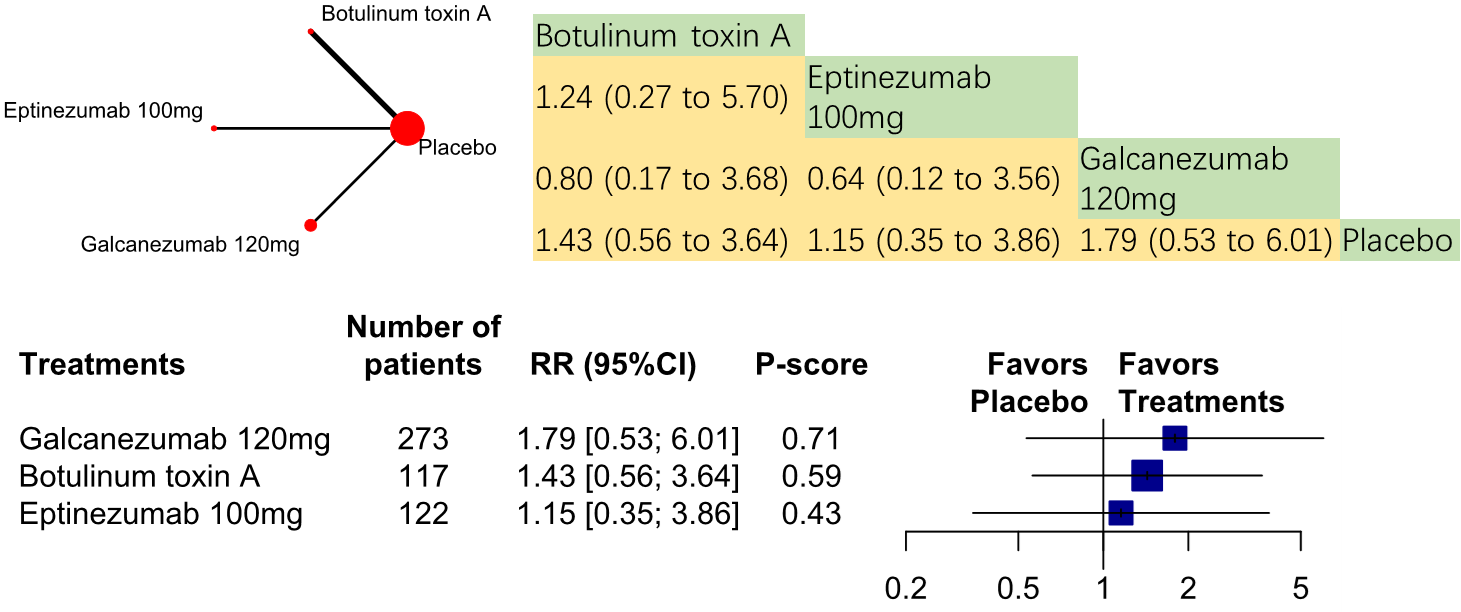


**Figure E3. Subgroup analysis in responder rate**

The subgroup analysis included the treatment arms of CGRPmAbs that adopted commercially available dosage and recommended treatment intervals (fremenezumab was administered quarterly at a dose of 675mg, galcanezumab was administered 120mg monthly, and eptinezumab was administered 100mg quarterly). The results showed that galcanezumab 120mg monthly was the most effective, but it was not significantly superior over eptinezumab and BoNT-A.

# Appendix F. Sensitivity analysis

**F1. Monthly headache days at week-12**


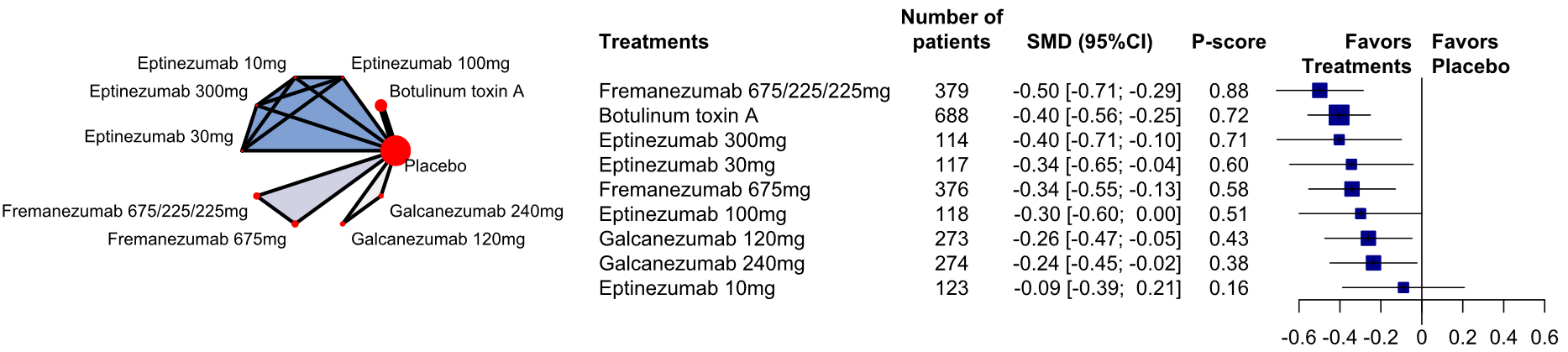


**Figure F1. Sensitivity analysis in monthly headache days**

The sensitivity analysis analysis included two BoNT-A trials and three CGRPmAbs trials that presented with similar baseline characteristics. The treatment with highest P-score has the highest possiblity to be the most effective treatment.

**F2. Monthly migraine days at week-12**


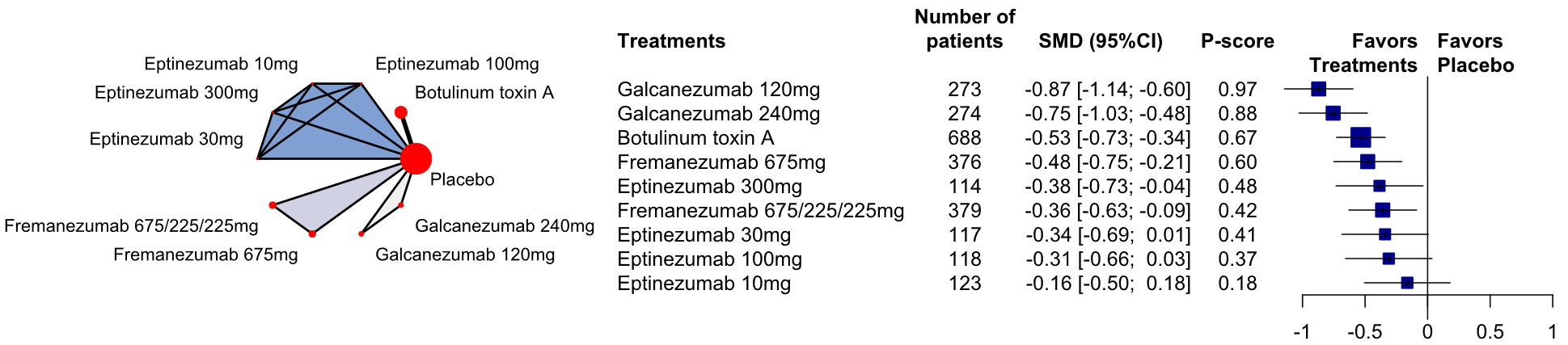


**Figure F2. Sensitivity analysis in monthly migraine days**

The sensitivity analysis analysis included two BoNT-A trials and three CGRPmAbs trials that presented with similar baseline characteristics. The treatment with highest P-score has the highest possiblity to be the most effective treatment.
